# Left Atrial Structure and Function in Heart Failure with Preserved Ejection Fraction: A RELAX Substudy

**DOI:** 10.1371/journal.pone.0164914

**Published:** 2016-11-03

**Authors:** Siddique A. Abbasi, Ravi V. Shah, Steven E. McNulty, Adrian F. Hernandez, Marc J. Semigran, Gregory D. Lewis, Michael Jerosch-Herold, Raymond J. Kim, Margaret M. Redfield, Raymond Y. Kwong

**Affiliations:** 1 Non-Invasive Cardiovascular Imaging Section, Brigham and Women’s Hospital, Boston, MA, United States of America; 2 Department of Medicine, Cardiology Division, Massachusetts General Hospital, Boston, MA, United States of America; 3 Duke Clinical Research Institute, Durham, NC, United States of America; 4 Duke Cardiovascular Magnetic Resonance Center, Duke University Medical Center, Durham, NC, United States of America; 5 Mayo Clinic Rochester, Rochester, MN, United States of America; Universita degli Studi di Napoli Federico II, ITALY

## Abstract

Given the emerging recognition of left atrial structure and function as an important marker of disease in heart failure with preserved ejection fraction (HF-pEF), we investigated the association between left atrial volume and function with markers of disease severity and cardiac structure in HF-pEF. We studied 100 patients enrolled in the PhosphdiesteRasE-5 Inhibition to Improve CLinical Status and EXercise Capacity in Diastolic Heart Failure (RELAX) trial who underwent cardiac magnetic resonance (CMR), cardiopulmonary exercise testing, and blood collection before randomization. Maximal left atrial volume index (LAVi; N = 100), left atrial emptying fraction (LAEF; N = 99; including passive and active components (LAEF_P_, LAEF_A_; N = 80, 79, respectively) were quantified by CMR. After adjustment for multiple testing, maximal LAVi was only associated with age (ρ = 0.39), transmitral filling patterns (medial E/e’ ρ = 0.43), and N-terminal pro-BNP (NT-proBNP; ρ = 0.65; all p<0.05). Lower LAEF was associated with older age, higher transmitral E/A ratio and higher NT-proBNP. Peak VO_2_ and V_E_/VCO_2_ slope were not associated with left atrial structure or function. After adjustment for age, sex, transmitral E/A ratio, CMR LV mass, LV ejection fraction, and creatinine clearance, NT-proBNP remained associated with maximal LAVi (β = 0.028, p = 0.0007) and total LAEF (β = -0.033, p = 0.001). Passive and active LAEF were most strongly associated with age and NT-proBNP, but not gas exchange or other markers of ventricular structure or filling properties. Left atrial volume and emptying function are associated most strongly with NT-proBNP and diastolic filling properties, but not significantly with gas exchange, in HFpEF. Further research to explore the relevance of left atrial structure and function in HF-pEF is warranted.

## Introduction

Heart failure with preserved left ventricular ejection fraction (HF-pEF) affects nearly 50% of all patients with HF. Given the clinical heterogeneity across patients with HF-pEF[1], structural markers that directly reflect the effects of chronic elevation in left ventricular (LV) filling pressure may provide additional characterization of the pathophysiology of HF-pEF. In this regard, left atrial volumes and emptying function have recently been identified as potentially useful prognostic markers in HF with reduced LV function[2], HF-pEF[3], and in pre-clinical HF states (e.g., diabetes)[4]. Left atrial volume may reflect the chronicity and severity of elevated left-sided filling pressure, and has been associated with natriuretic peptides[5] and outcomes in different HF states [3]. Characterizing left atrial structure and emptying function in HF-pEF remains an area of active interest.

Here, we sought to investigate the relationship between left atrial structure and function with markers of fibrosis, hemodynamic stress, and prognostic indices of gas exchange in HF-pEF. We hypothesized that a greater left atrial volume and lower left atrial emptying function would be associated with decreased peak aerobic capacity, less ventilatory efficiency, and increased circulating biomarkers of fibrosis and N-terminal pro-BNP in HF-pEF. To address this hypothesis, we calculated left atrial volumes and emptying function by cardiac magnetic resonance (CMR) in HF-pEF patients in the Phosphodiesterase-5 Inhibition to Improve Clinical Status and Exercise Capacity in Heart Failure with Preserved Ejection Fraction (RELAX) study. Ultimately, we sought to provide further evidence of the physiologic importance of left atrial structure in cardiopulmonary structure and fitness in HF-pEF.

## Materials and Methods

### Patient population

The details of the RELAX trial have been previously reported[6]. RELAX was a National Heart, Lung, and Blood Institute-sponsored, multisite randomized clinical trial of phosphodiesterase-5 inhibition with sildenafil to improve exercise capacity in patients with HF with preserved LV ejection fraction. Participants with a normal LVEF (>50%) and stable clinical HF (New York Heart Association class II-IV) were eligible if they had impairment in functional capacity, as defined by: peak oxygen consumption VO_2_ on cardiopulmonary exercise testing (CPET) less than 60% predicted for age- and sex-normative values (with adequate effort) and either (1) elevated N-terminal pro-B-type natriuretic peptide (NT-pro BNP) ≥ 400 pg/ml or elevated LV pulmonary capillary wedge pressure (>20 mmHg at rest or >25 mmHg during exercise). Patients were excluded if there was clinical evidence of myopericardial disease (e.g., infiltrative, inflammatory, or hypertrophic), pulmonary arterial hypertension, recent active coronary disease (e.g., revascularization within 60 days), or alternative, non-cardiac causes of dyspnea (e.g., significant lung disease or morbid obesity by clinical evaluation); a full list of exclusion criteria is available[6]. The primary endpoint was change in peak VO_2_ over 24 weeks of therapy. In addition to clinical evaluation (CPET testing, venous blood collection, 6-minute walk testing, echocardiography), cardiac magnetic resonance (CMR) imaging was performed on a subset of patients within RELAX who satisfied the main trial inclusion criteria and: (1) no implanted non-MRI safe device, (2) no claustrophobia, (3) able to lie flat and breath-hold for at least 15 seconds, (4) adequate size for magnet bore, and (5) not in atrial fibrillation. Of the 216 patients randomized in RELAX, 117 (54%) were eligible for CMR imaging. The details of echocardiography, CPET testing, biomarker assays, and clinical end-points are described in the main RELAX manuscript[6]. All study participants provided written informed consent prior to screening. The National Heart, Lung, and Blood Institute–sponsored Heart Failure Clinical Research Network conceived, designed, and conducted the RELAX trial. The trial protocol was approved by a National Heart, Lung, and Blood Institute–appointed protocol review committee and data and safety monitoring board, and by the institutional review board at each participating site.

### RELAX cardiac magnetic resonance imaging protocol

CMR imaging was performed using 1.5 Tesla scanners from all major vendors (e.g. Siemens, General Electric, Phillips). The CMR protocol consisted of (1) conventional scout images for cardiac localization; (2) a complete stack of LV short-axis cine images for LV mass and function using a balanced steady state free precession technique (SSFP; temporal resolution 35–40 msec, slice thickness 6 mm with 2 mm gap, flip angle ~60°, typical in-plane resolution 1.5 x 1.9 mm; (3) SSFP cine imaging of the tubular ascending aorta for measurement of aortic distensibility (cross-sectional plane perpendicular to the tubular ascending aorta approximately 4 cm above the aortic valve using the same sequence parameters as in step 2). Contrast was not administered routinely as prescribed by the RELAX protocol. Analysis of left ventricular function, volumes, and mass was performed on using QMass 4.2 (Medis, Leiden, The Netherlands) by a Simpson’s approximation (standard Society of Cardiovascular Magnetic Resonance published guidelines[7]). Aortic distensibility was calculated using blood pressure and heart rate measured at the time of CMR acquisition, as previously described[8]. Left atrial volumes and function were indexed to body surface area and analyzed as described below.

### Measurements of left atrial volumes and function

**Fig 1:**
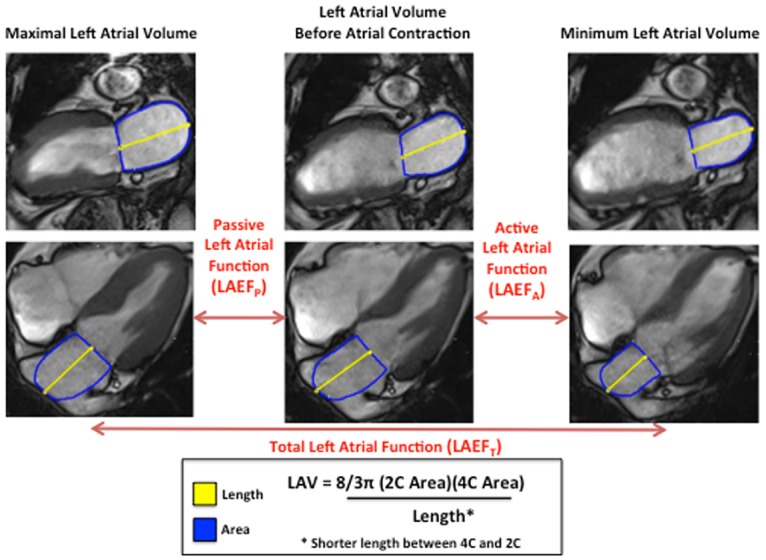
Quantification of left atrial volumes and left atrial function. Cine steady state free precession images are shown in the two- and four-chamber views at the point of maximum left atrial volume, before atrial contraction, and minimum left atrial volume. Length = Yellow, Area = Blue.

Left atrial volumes were calculated at the agreement of three observers (SAA, RVS, RYK) using the 2- and 4-chamber cine SSFP sequences by way of the biplane area-length method: LA volume = (8**π/3)** X (Area_4-chamber_) X (Area_2-chamber_) / atrial length, using the shorter atrial length from either the 4- or 2-chamber view. Left atrial volumes were calculated at the end of ventricular systole (LAV_MAX_), just before atrial contraction (LAV_BAC_), and at the end of ventricular diastole (LAV_MIN_). All left atrial volumes were indexed to body surface area (LAVi). Finally, left atrial function was quantified into its component parts: 1) Passive left atrial emptying fraction (LAEF_P_) = (LAV_MAX_−LAV_BAC_) X 100%/ LAV_MAX_; 2) Active left atrial emptying fraction (LAEF_A_) = (LAV_BAC_−LAV_MIN_) X 100%/ LAV_BAC_; and 3) Total left atrial emptying fraction (LAEF) = (LAV_MAX_−LAV_MIN_) X 100%/ LAV_MAX_ (Fig 1). Left atrial area measurements were obtained by manual planimetry using commercially available post-processing software packages (CVI42, Circle Cardiovascular Imaging).

### Statistical analysis

Continuous data were summarized using medians and interquartile ranges, and compared between groups using a Wilcoxon rank-sum test. Categorical data were summarized using percentages, and compared between groups using chi-square tests. Our primary outcome was the association of LAVi and total LAEF with two measures of gas exchange (peak VO_2_, V_E_/VCO_2_ slope) and biomarkers reflecting fibrosis and hemodynamic stress (NT-proBNP, galectin-3, PIIINP). Our secondary outcome was the association of LAVi and LAEF with selected imaging parameters physiologically central to greater left atrial pressures in HF-pEF (age, LV mass index, transmitral echo Doppler indices, aortic distensibility). In a subset of individuals with available measures, we examined associations with components of left atrial emptying (LAEF_A_, LAEF_P_). Spearman bivariate correlation coefficients were estimated to measure the strength of association for primary and secondary outcomes. For those primary outcome measures that were significant in bivariate correlation (peak VO_2_, V_E_/VCO_2_, and NT-proBNP), we constructed multivariable linear models to test for association between LAVi, LAEF, and its components with each outcome, adjusted for age, gender, CMR LV mass and LV ejection fraction, echocardiographic E/A ratio, and creatinine clearance (all measures of disease severity or potential confounders). NT-proBNP was log-transformed for analysis to establish linearity. Model residuals were examined to ensure no departures from linearity. Given the multiple comparisons performed, we used a Bonferroni type 1 corrected p-value for multiple comparisons. SAS version 9.2 (SAS Institute, Cary, NC) was used for all analyses.

## Results

### Baseline characteristics

Baseline characteristics of RELAX patients undergoing CMR with analyzable left atrial data (N = 100) and patients who did not undergo CMR (N = 99) are shown in Table 1. Of the 117 subjects undergoing CMR in RELAX, 100 (85%) had analyzable data for left atrial volumes, with the remainder unanalyzable due to absence of suitable or required images for left atrial volume assessment (N = 17; 2- and 4-chamber). In general, the overall study cohort was similar to other studies in HF-pEF, with a median age 67 years, 50% male, and obese (median body mass index 33 kg/m^2^) with decreased cardiorespiratory fitness (median peak VO_2_ 12.3 ml/kg/min). Of note, patients who did not have a CMR performed in RELAX were older (p = 0.02), with significantly higher NT-proBNP (p<0.0001), galectin-3 (p = 0.005) and lower peak VO_2_ (p = 0.03). Of the 100 individuals who had analyzable left atrial volumes by CMR, the majority had total left atrial emptying function (LAEF, our primary endpoint, N = 96), while active and passive left atrial emptying function was assessable in a majority (LAEF_A_, N = 79; LAEF_P_, N = 80). Unanalyzable images for components of atrial function were due to inability to determine precise phase of atrial contraction. CMR characteristics of the study population are shown in Table 2. LV ejection fraction (by definition) was preserved in these individuals (median LVEF 65.6%).

**Table 1:** Clinical characteristics of RELAX subjects with and without CMR imaging at study entry. Continuous variables are expressed as median (interquartile range).

Baseline Variable	LA volume assessed (N = 100)	No CMR performed (N = 99)	P value (Wilcoxon or Chi-Square)
Age	67 (61, 73.5)	70 (64, 79)	0.02
Gender			
Male	50 (50%)	58 (58.6%)	0.22
Female	50 (50%)	41 (41.4%)	
Number of HF Hospitalization in Prior Year			
0	62 (62%)	59 (59.6%)	0.36
1	33 (33%)	24 (24.2%)	
2	2 (2%)	7 (7.1%)	
3	1 (1%)	3 (3%)	
4+	2 (2%)	6 (6.1%)	
Hypertension (%)	86 (86%)	83 (83.8%)	0.67
Diabetes (%)	44 (44%)	45 (45.5%)	0.84
Etiology of Heart Failure			
Ischemic	34 (34%)	43 (43.4%)	0.17
Non-ischemic	66 (66%)	56 (56.6%)	
Systolic BP (mmHg)	128 (113, 139.5)	122 (112, 137)	0.32
Body mass index (kg/m^2^)	33 (28, 40)	33 (28.5, 38.6)	0.89
Creatinine clearance (Cockcroft-Gault; ml/min)	71 (53.8, 110.8)	66.1 (51.8, 88.9)	0.23
Medial e’ (m/sec)	0.06 (0.05, 0.07), N = 92	0.06 (0.05, 0.08), N = 88	0.25
Lateral e’ (m/sec)	0.08 (0.06, 0.10), N = 91	0.09 (0.07, 0.11), N = 84	0.03
Medial E/e'	16.0 (11.3, 20), N = 89	16.2 (12.9, 25), N = 82	0.13
Lateral E/e'	11.4 (8.7, 15.7), N = 88	13.2 (8.4, 17.3), N = 80	0.55
Pulmonary arterial systolic pressure (mmHg)	42.4 (32.5, 49.8), N = 56	44.3 (34.2, 53.6), N = 70	0.48
Peak VO_2_ (ml/min/kg)	12.3 (10.5, 15.1), N = 99	11.4 (10.1, 13.4), N = 99	0.03
V_E_/VCO_2_ slope	32.4 (27.2, 37), N = 96	33.7 (29.6, 39.6), N = 98	0.08
N-terminal pro-B-type natriuretic peptide (pg/ml)	457.5 (108.2, 1266), N = 98	1129 (633.1, 1880), N = 98	<0.0001
Galectin-3 (ng/ml)	13.1 (10.5, 16.9), N = 98	15.1 (12.5, 19.9), N = 93	0.005
Pro-collagen III N-terminal propeptide (μg/l)	7.6 (5.6, 10), N = 99	8 (6.3, 11), N = 98	0.30

Abbreviations: LV, left ventricular; HF, heart failure; CMR, cardiac magnetic resonance; BP, blood pressure.

**Table 2:** Baseline left ventricular and left atrial CMR characteristics.

Baseline Variable*	Value
CMR left ventricular mass (grams)	139.5 (107.7, 174.5)
CMR left ventricular ejection fraction (%)	65.6 (57.5, 69.2)
CMR left ventricular mass index (grams/m^2^)	63.4 (54.7, 76.7)
Maximal left atrial volume index (ml/m^2^)	46.4 (34.7, 55.2)
Total left atrial emptying fraction (%), N = 96	43.6 (32, 53.8)
Active left atrial emptying fraction (%), N = 79	34.4 (23.2, 42)
Passive left atrial emptying fraction (%), N = 80	17.5 (12.6, 23.9)

*Number of observations for RELAX participants specified in text.

Abbreviations: CMR, cardiac magnetic resonance. All CMR indices are indexed to body surface area. LV papillary muscle mass *not included* as part of total left ventricular mass.

### Left atrial volume and total emptying function is associated with N-terminal pro-BNP and age, but not with gas exchange

**Fig 2:**
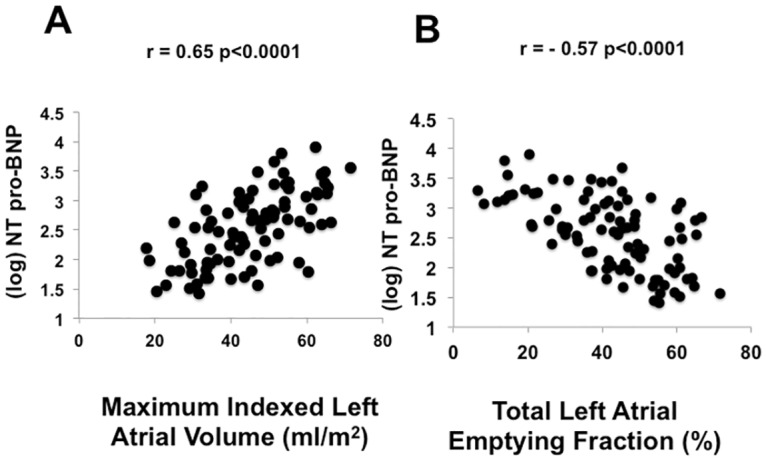
Relationship of NT-pro-BNP with (A) maximum left atrial volume index and (B) total left atrial emptying fraction. Spearman correlation coefficient is reported.

Unadjusted (Spearman) associations between maximal left atrial volume and left atrial function are shown in Table 3 (and Fig 2). After adjustment for multiple hypothesis testing, maximal LAVi was associated with age (ρ = 0.39, p<0.0001), and echocardiographic and biochemical evidence of higher LV filling pressure (by E/e’ ratio and NT-proBNP), but not gas exchange. Similarly, a lower LAEF (decreased left atrial contractile function) was associated with older age, higher transmitral E/A ratio and NT-proBNP (Fig 2), but not gas exchange. After adjustment in linear models, the association between LAVi or LAEF and NT-proBNP remained significant, with a greater LAVi (β = 0.028, p = 0.0007) and lower LAEF (β = -0.033, p = 0.001) independently associated with NT-proBNP. Of note, neither LAEF nor LAVi were associated with circulating markers of collagen turnover (pro-collagen telopeptides) or fibrosis (galectin-3). In the subgroup with components of LAEF available (active and passive), we found associations between lower LAEF_P_ with older age and both LAEF_A_ and LAEF_P_ with higher NT-proBNP, but not with gas exchange metrics; only the association between NT-proBNP and LAEF_A_ withstood multivariable adjustment (β = -0.032, p = 0.005).

**Table 3:** Spearman correlation coefficients in RELAX subjects for left atrial volumes or function and indices of hemodynamic stress, left ventricular remodeling, diastolic function, and gas exchange.

	LAV Max indexed	LAEF Total	LAEF Active	LAEF Passive
**Age**	0.39*	-0.39*	-0.23	-0.45*
**Imaging indices**				
LV mass index (CMR)	0.23	-0.02	-0.04	-0.19
Aortic distensibility	-0.26	0.31	0.29	0.35
Medial E/e’ ratio	0.43*	-0.28	-0.30	-0.10
Lateral E/e’ ratio	0.33*	-0.25	-0.26	-0.16
Transmitral E/A ratio	0.02	-0.33*	-0.28	-0.26
**Biomarkers**				
NT-pro BNP	0.65*	-0.57*	-0.49*	-0.34*
Galectin-3	0.12	-0.15	-0.09	-0.12
PIIINP	0.10	-0.12	-0.14	-0.10
**Gas exchange**				
Peak VO_2_	-0.22	0.22	0.19	0.06
V_E_/VCO_2_ slope	0.23	-0.26	-0.20	-0.22

Abbreviations: CMR, cardiac magnetic resonance, LV, left ventricular, NT-proBNP, N-terminal pro-B-type natriuretic peptide. Given 11 total comparisons per left atrial measure, we used a Bonferroni corrected p-value (0.05/11 = 0.0045) as significant.

* Denotes all significant p-values.

## Discussion

In this report, we describe the distribution of left atrial volumes and function by cardiac magnetic resonance in a carefully phenotyped, prospective population of patients with HF-pEF. We found that left atrial volume and function were most strongly associated with circulating N-terminal pro-BNP concentrations, and more modestly with age and transmitral LV filling patterns. Importantly, after correction for multiple hypothesis testing, we did not observe an association between left atrial emptying function or left atrial volume with gas exchange. Associations between NT-proBNP and left atrial structure and function were robust to adjustment. These findings suggest that left atrial structure and function most closely reflect hemodynamic stress and remodeling in HF-pEF (by NT-proBNP and diastolic filling properties), but are not directly associated with prognostic measures of gas exchange.

The recognition of a role for left atrial structure and function in HF has undergone a renaissance[9]. The role of left atrial function in HF-pEF is particularly relevant, given underlying myocardial hypertrophy, fibrosis, and impaired relaxation that is thought to characterize HF-pEF[10]. Published work in HF-pEF has focused on the prognostic utility of left atrial size[11,12] or the role of left atrial size and function in associated comorbid illnesses (e.g., atrial fibrillation[13,14] or diabetes[4]). Until recently, little published work has focused on invasive and non-invasive characterization of left atrial structure and function in HF-pEF.

One unanticipated finding from our analysis was the lack of association between left atrial emptying function or left atrial volume with measures of gas exchange. Given the relationship between peak VO_2_ and natriuretic peptides in patients with a variety of cardiopulmonary diseases [15,16], our expectation was to have found an association between the left atrium and peak VO_2_ in patients with HF-pEF. One possible explanation for this finding was the exclusion of patients with atrial fibrillation (AF) in the current analysis. Indeed, prior work from RELAX had demonstrated that individuals with AF (which represented nearly 40% of the total study population) had significantly lower peak VO_2_ [17]. Further investigation—inclusive of patient with AF—is therefore warranted to better understand the relationship between ventilatory efficiency and left atrial structure and function. Additionally, the study may have been underpowered (n = 100) to detect such an association between atrial characteristics and peak VO_2_.

Recent work from Melenovsky and colleagues investigated 198 patients (51% with HF-pEF) with integrated hemodynamic and echocardiographic measures of left atrial structure and function[3]. These investigators found that at similar LA pressures, patients with HF-pEF exhibited higher peak LA pressure, higher LA stiffness and pulsatility, relative to patients with HF with reduced LV function. Impairment in LA function was associated with right ventricular dysfunction and higher pulmonary vascular resistance. Finally, greater total left atrial function was associated with lower mortality in HF-pEF. When compared with this recent study, we observed a similar range of LA volume, total LA emptying function, and active LA emptying fraction by CMR (Melenovsky study: LA volume: 41±10 ml/m^2^; total LAEF 39±17%; active LAEF: 30±14%); RELAX study participants had a lower passive LAEF relative to the Melenovsky study (26±9%). Our study additionally delineated association between left atrial structure and function with age and NT-proBNP, concordant with the invasive hemodynamic findings of Melenovsky et al. Taken together, these results suggest that impairment in left atrial structure and function (reflecting increased hemodynamic stress and higher LV filling pressure) is prevalent and plays an important role in HF-pEF physiology. Our study further demonstrates that CMR methods to interrogate the left atrium may provide a useful tool to non-invasively investigate HF-pEF physiology.

The conclusions of our study must be viewed in light of its design. Our study is small relative to other community-based studies of left atrial volume and function, which may contribute to modest effect sizes, lack of association after multivariable adjustment, and the potential for type 1 error with the multiple correlations investigated. However, the RELAX study population is rigorously selected for HF-pEF, which is unique, and the original study was not powered on left atrial imaging endpoints. Our results therefore may motivate additional studies utilizing this parameter as a physiologic endpoint. We recognize that mitral valve function was not quantified in CMR acquisition; however, individuals with severe valvular heart disease (mitral or aortic stenosis or regurgitation by echocardiography) were excluded from RELAX. Given the limited event rate and follow-up in RELAX, we did not report long-term rates of HF hospitalization or mortality in our cohort, which also merits investigation in larger, prospective studies of individuals with HF-pEF.

## Conclusion

In conclusion, in a well-phenotyped cohort of patients with HF-pEF, left atrial volumes and function are associated with aging, echocardiographic diastolic filling properties, and NT-proBNP. Gas exchange indices were not associated with left atrial structure or function. These results suggest that the left atrium may be a marker of hemodynamic stress and neurohormonal activation in a carefully phenotyped group of patients with HF-pEF. Further research in larger populations is warranted to investigate the role of left atrial morphology and function in cardiorespiratory fitness, dyspnea generation, and outcome in HF-pEF.
